# Inter-rater reliability and validity of good pharmacy practices measures in inspection of public sector health facility pharmacies in Uganda

**DOI:** 10.1186/s40545-018-0161-y

**Published:** 2019-01-22

**Authors:** Brian Sekayombya, David Nahamya, Laura Garabedian, Morries Seru, Birna Trap

**Affiliations:** 1 Management Sciences for Health, Plot 15, Princess Anne Drive, Bugolobi, P.O. Box 71419, Kampala Uganda; 2 National Drug Authority, Plot 46 48 Lumumba Avenue, Kampala, Uganda; 3 Harvard Pilgrim Health Care Institute, 401 Park Drive, Suite 40, Boston, MA 02215 USA; 4 Ministry of Health, Pharmacy Division, Lourdel Road, Wandegeya, Kampala Uganda

**Keywords:** Good pharmacy practices, Validity, Inter-rater reliability, Facility inspection, Indicators, Good pharmacy practices measures, Data quality audit, Inspection quality

## Abstract

**Background:**

The National Drug Authority (NDA) inspects and certifies private and public sector pharmacies in Uganda using an indicator-based inspection tool that measures adherence to good pharmacy practices (GPP). 67 measures identify the situation in the domains of premises, dispensing quality, stores management, and operating requirements. Although the GPP measures are well-recognized and used internationally, little is known about their validity and reliability. The study aimed to assess validity, which measures agreement of GPP measures between a gold standard inspector and NDA inspector and inter-rater reliability (IRR), which measures agreement among NDA inspectors, of GPP measures.

**Methods:**

We assessed validity and IRR by four teams of inspectors in eight government health facilities that represent three levels of care. Each team inspected two facilities, resulting in 24 total inspections. Each team comprised one central-level inspector, one district-level inspector, and one gold-standard inspector (i.e., a very experienced central-level inspector). We calculated median validity and IRR for each GPP measure, overall, indicator categorized as either critical, major, or minor, by domains, by the inspection decision (i.e., certified or not certified) and by adequatevalidity and IRR score (i.e., score ≥ 75%).

**Results:**

The median validity for all GPP measures was 69%, with 29 (43%) measures having an adequate validity of ≥75%. The median IRR for all GPP measures was 71%, with 31 (46%) having an adequate IRR measure of ≥75%. Validity did not differ significantly by indicator category, domain or level of care. Adequate IRR and validity score (≥75%) was lowest for critical measures, which are key determinants of the certification decision, scoring 20 and 40% respectively. District inspectors had lower median validity for critical indicators and premises and higher validity for store management. Compared to central inspectors, the validity of district inspectors’certification decisions was lower; in the eight facilities, three district inspectors agreed with gold standard inspector vs. all eight central inspectors.

**Conclusions:**

Our findings question the validity and reliability of many GPP inspection measures, particularly critical measures that greatly impact certification decision. This study demonstrates the need for assessments of, and interventions to improve, validity and reproducibility of GPP measures and inspections.

**Electronic supplementary material:**

The online version of this article (10.1186/s40545-018-0161-y) contains supplementary material, which is available to authorized users.

## Background

The National Drug Authority (NDA), established in 1993, plays a critical role in ensuring that quality and efficacious medicines are available in Uganda. Since its inception, NDA has inspected and certified pharmaceutical outlets in the private sector; in 2013, NDA started carrying out good pharmacy practice (GPP) inspections and certifications in public sector medicines outlets. The inspections are intended to ensure a minimum standard of the condition of storing, handling, and dispensing medicines at the health facility that are based on World Health Organization/International Pharmaceutical Federation Good Pharmacy Practices standards [1]. Trained NDA inspectors measure adherence to GPP standards using an indicator-based inspection tool that was grounded in international standards but adapted to fit the local context and to clarify what needed to be measured. If minimum standards are met, the facility is certified [2].

Ensuring that GPP inspections are valid (i.e., they produce accurate results), and reliable (i.e., they produce consistent results), is important to public health. Too often, however, well-known and frequently used indicator-based measures, such as the World Health Organization rational drug use indicators, are used without investing the time and effort to assure the quality of data collection and interpretation or to assess data quality or measure reproducibility [3–5].

To prepare the public sector health facility pharmacies to build the necessary medicines management capacity to meet GPP standards, the Ministry of Health adopted a supportive supervision, performance assessment, and recognition strategy (SPARS) that is implemented by trained district supervisors using an indicator-based assessment tool [6]. The Uganda Ministry of Health prioritized efforts to assure SPARS data reliability, because the information would be used to make programmatic and policy decisions for the pharmaceutical sector. A study assessing agreement of measurements by different supervisors found that the mean inter-rater reliability (IRR) of the SPARS measures was initially only 57% [7]. Given the initially poor IRR of the SPARS measures and the overlap between SPARS and GPP assessment tools (73% of GPP inspection measures are also SPARS measures), assessing the reliability of GPP measures and certifications was important. Furthermore, our study is the first to assess IRR and validity of GPP measures.

This study aims to determine validity and IRR of the GPP measures and validity of the overall certification decision.

## Methodology

We used a cross-sectional design to assess the reliability of GPP inspections by comparing the GPP measures between central and district inspectors and the validity of GPP inspections by comparing GPP measures and certification decisions between the inspectors and a gold standard inspector.

### Setting

In Uganda, health care services are delivered by the government, private not-for-profit,and private for-profit sectors. Within the government and private not-for-profit sectors, levels of health care delivery include health centres (HC) 1, 2, 3, and 4, general hospitals, and regional/national referral hospitals. Each facility level varies by population served, staffing, infrastructure, services, and patient load. There are just over 4000 public sector health facilities that dispense medicines and are therefore required to be certified by the NDA as adhering to GPP standards. HC 1 level represents the village health worker program, which is not included in the GPP program.

### Selection methods

#### Study inspectors

Based on a list of 41 NDA inspectors who had conducted 10 or more inspections in pharmacies or drug shops, we grouped inspectors into two categories: central-level inspectors (*n* = 12) or district-based inspectors (*n* = 29). We excluded 15 district inspectors who had received extra training as medicines management supervisors in the SPARS program. From the remaining 26 inspectors, we randomly selected four inspectors from each group to create four pairs each comprising one central and one district inspector.

#### Gold standard inspector

The gold standard inspector was a senior central-level inspector with a good understanding of the GPP measures and extensive experience in inspection in general and use of the GPP tool, in particular. The same gold standard inspector accompanied all four teams of inspectors. He conducted an independent inspection at the same time the teams carried out their inspections, but otherwise, he did not influence them.

#### Districts and facilities

At the time of this study, GPP inspection and certification were already being implemented in public sector health facilities. Initial GPP inspections found that lower levels of care, HC 2, HC 3, and HC 4, had lower certification rates (52, 60, 53%, respectively) compared to hospitals (90%) [2]. It is important to note that differences in infrastructure, staffing, and patient load between health centres and hospitals likely affected the results, so we decided to include only health centres 2–4 in this study.

We purposefully selected the central region to simplify logistics and then chose three districts (of 24) that met inclusion criteria as follows:

- Had at least four lower-level facilities that had not been previously inspected
- Facilities had received at least four SPARS visits in preparation for GPP inspection
- None of the selected district-level inspectors resided in or had jurisdiction over the district
- Close to each other

We randomly selected eight facilities from the three districts: three HC 2, three HC 3, and two HC 4 facilities. Each team assessed two facilities in the same district on the same day.

### Inspection tool and classification of measures

**Fig. 1:**
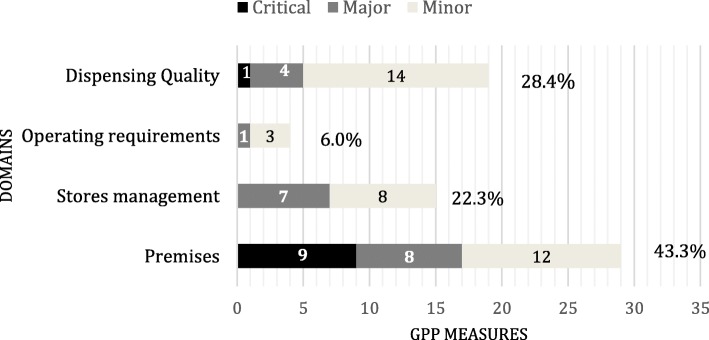
Classification of GPP measures by domains and categories (critical, major and minor GPP measures)

The GPP inspection tools for public sector and private sector pharmacies are largely similar. The GPP inspection tool for the public sector, which was used in this study, is presented in Additional file 1. To fill in the tool, the inspectors collect retrospective and prospective data using record reviews, direct observations, and questions. The inspectors collected information on 67 GPP measures, not including general administrative information such as staffing. The GPP measures are listed in Additional file 2. The measures are classified as critical, major, and minor and cover four domains: premises, stores management, operating requirements, and dispensing quality (Fig. 1). Most GPP measures assess performance in either the store or the dispensary, but about one-third measure performance at both locations, counted as two measures. The measures were also classified as either objective (42) or subjective (25), which require personal judgement. The type of measure is given in Additional file 2.

About 80% of the GPP measures are scored as categorical three-point variables (acceptable, needs improvement, or unacceptable), and about 20% are scored as two-point yes or no variables.

Acceptable, Needs Improvement, or Yes are passing scores for certification decision. To become GPP certified, facilities must pass all critical measures and more than half of major measures.

The study inspections took place over four days, with each team (consisting of one district inspector, one central level inspector, and one gold standard inspector) performing two inspections each day for a total of 24 assessments at eight facilities (Table 1).

**Table 1:** Implementation plan for inspections with inspectors, facilities, and assessments

Team A			Team B	Team C	Team D
District Inspector (DI) 1			DI 2	DI 3	DI 4
Central Inspector (CI) 1			CI 2	CI 3	CI 4
Day	Facility	Team	Gold standard (GSI)	Assessments	
1	1	Team A	GSI 1	3	
1	2	Team A	GSI 1	3	
2	3	Team B	GSI 1	3	
2	4	Team B	GSI 1	3	
3	5	Team C	GSI 1	3	
3	6	Team C	GSI 1	3	
4	7	Team D	GSI 1	3	
4	8	Team D	GSI 1	3	
Total				24	

Inspectors received a briefing on the study purpose and how the team inspections would be conducted and were trained in how to use the electronic data collection form. For measures that required selecting patients or patient records, the gold standard inspector ensured that the inspectors reviewed the same records and patients as follows:

- The gold standard inspector selected three patients from each facility for the study inspectors to each interview independently to assess patients’ knowledge of how to take medicines. One by one, the two study inspectors interviewed the patients and assessed his or her knowledge without the other inspector overhearing the interview. The gold standard inspector would not interview the patients himself but would listen to the two interviews of the same patient and determine the “gold” response without interviewing the patient a third time.
- To ensure that all inspectors assessed the same medicine label, the gold standard inspector selected the medicine containers from the medicines dispensed to the patients, then all three inspectors independently assessed the quality of the labelling for each of the selected medicine containers.
- To ensure that all three inspectors assessed the same medicines and records (e.g., stock cards), the gold standard inspector identified which medicines to evaluate related to inventory and storage management.

On arrival at the health facility, the two study inspectors and the gold standard inspector informed the facility in charge about the NDA-mandated inspection and explained the process. Data was initially collected using a standardized paper-based tool. Each inspector entered the manual data into an electronic GPP data collection tool at the end of the day, and the data was exported to Excel and subsequently to STATA for analysis.

### Data analysis

As noted above, validity measures percent agreement between each inspector and the gold standard, while IRR measures percent agreement between the district and central inspectors.

#### IRR score

IRR was scored 100% when both the district and central inspectors gave the same score. The gold standard inspector is not included in the IRR calculation. For a two-point measure (yes or no), both inspectors either scored “yes” or both scored “no”. For a three-point measure (acceptable, needs improvement, or unacceptable), both inspectors either scored “needs improvement” or both scored “acceptable” or both scored “not acceptable”. The IRR of a GPP measure is the sum of the number of times both study inspectors agreed divided by the number of facilities (i.e., 8). The IRR result is given as a percentage. The overall IRR score is calculated as the median IRR for all 67 measures. The IRR score for the three categories— critical indicators (*n* = 10 measures), major indicators (*n* = 20 measures) and minor indicators *n* = 37 measures) is calculated by taking the median IRR score for the number of measures within the category. The IRR score for a domain—premises (*n* = 29 measures), dispensing practices (*n* = 19 measures), stores management (*n* = 15 measures), and operations (*n* = 5 measures) is calculated be taking the median IRR score for the number of measures within each domain. The calculation is similar for level of care, such as HC 2 facilities (*n* = 3), HC 3 facilities (*n* = 3) and HC 4 (*n* = 2); that is, the median IRR score is taken for the measures within each level of care. The IRR score for objective (*n* = 42) and subjective (*n* = 25) measures is calculated as the median for objective and subjective measures.

#### Validity score

Validity compares the score of each inspector (central and district) independently to the score of the gold standard inspector. If an inspector gives the same score as the gold standard (for either two-point or three-point measures), the validity score for that inspector is 100% and if there is disagreement it is 0%. To calculate the facility validity score for a GPP measure, the validity score is the average score of both inspectors: 0% if neither of the two inspectors was in agreement with the gold standard inspector, 50% if one inspector is in agreement with the gold standard and the other not, and 100% if both inspectors are in agreement with the gold standard. The validity score for each GPP measure is calculated by taking the mean of the facility validity scores for the measure. We also present the median validity score overall (i.e., for all measures at the eight facilities), for district inspectors overall and central inspectors overall, and by indicator categories, type (objective or subjective), domains, and level of care.

Table 2 shows an example of the data table and IRR and validity calculations for a GPP measure by level of care and if the measure is adequate (i.e., ≥75%).

**Table 2:** Mean IRR and validity calculations for one binary (yes or no) GPP measure

Facility/Level	Study Inspector a	Study Inspector b	Gold Standard	IRR*	Validity
1 – HC2	1	0	1	0/1 (0%)	1/2 (50%)
2 – HC2	0	1	1	0/1 (0%)	1/2 (50%)
3 – HC2	0	0	0	1/1 (100%)	2/2 (100%)
4 – HC3	1	0	1	0/1 (0%)	1/2 (50%)
5 – HC3	1	1	1	1/1 (100%)	2/2 (100%)
6 – HC3	0	0	0	1/1 (100%)	2/2 (100%)
7 – HC4	1	1	0	1/1 (100%)	0/2 (0%)
8 – HC4	0	0	0	1/1 (100%)	2/2 (100%)
IRR and Validity score for a single measure				62.5% (500/8)	68.8% (550/8)
≥75%				No (0)	No (0)
HC2				1/3 (30%)	4/6 (67%)
HC3				2/3 (67%)	5/6 (83%)
HC4				2/2 (100%)	2/4 (50%)

#### “Adequate” IRR and validity scores

IRR score and validity scores were deemed adequate if they were ≥ 75%, following a rule of thumb for adequate reliability [8]. IRR and validity scores between 50 and 75% were moderately adequate, and those < 50% were considered to be inadequate in this analysis.

#### Validity of certification decisions

The facility certification decision was determined based on failed critical and major measures. The validity of the certification decision was calculated as the percent agreement between the gold standard and each study inspector, stratified by central versus district inspector.

#### Statistical analysis

We calculated median scores and percentage of scores that met the ≥75% threshold for IRR and validity overall, for each GPP measure, and by measure category (i.e., critical, major, and minor), type (objective and subjective), domain, and facility level as well as between central inspectors and district inspectors (for validity only). We used Wilcoxon signed rank test to compare median validity and IRR scores overall and by category, type, domain, and level of care. Similar tests were used to compare median validity scores between central and district inspectors. We used logistic regression to assess the association between adequate IRR and validity scores with indicator categories, domain, and level of care. All these analyses were conducted using STATA 13 and Excel 2013. In the results, we only show *p*-values when significant (i.e., *p* < 0.05).

#### Problems Encountered with Missing Measures

The total number of assessments by the three inspectors in the eight facilities should have included 1608 individual GPP measures. However, for various reasons, some inspectors missed assessing one GPP measure, or in some cases, all three inspectors missed the assessment. At one facility, the store was locked, which made the assessment of 23 measures by all three inspectors impossible, and at another facility, there were no patients to interview, so the three inspectors could not obtain patient-dependent GPP measures. Some of these missing assessments included critical indicators. In total, 178 (11.1%) of the assessments of GPP measures were missing, of which 26 (10.8%) were critical. If one central or district inspector missed a GPP measure, the IRR could not be calculated for that measure at that facility, and the GPP measure was averaged for only seven facilities. Validity was calculated based on only one inspector versus the gold standard inspector result and averaged for eight facilities. If the gold standard inspector had no assessment for a measure at one facility, the IRR was calculated, but the validity for the GPP measure was based on seven facilities. The certification decision was calculated by scoring the missing critical indicators as passed. When all three inspectors missed the measure, neither IRR or validity could be calculated for that facility, and the GPP measure was calculated based on seven facility scores.

## Results

Table 3 includes median IRR and validity scores and the percent of indicators that achieved adequate score (≥75%), overall, and by indicator categories, domains, and level of care. Table 3 also provides validity scores stratified by district and central level inspectors. Mean validity and IRR scores for all GPP measures and for adequate measures is given in Additional file 2.

**Table 3:** Number and percentage of GPP measures with overall median and adequate (> 75%) mean scores for validity and IRR and validity for inspector type, category, domain, and level of care

Validity					IRR				Validity						
									Central level Inspector			District based inspector			
Median GPP scores															
	n	median	IQR	p-values	n	median	IQR	*p*-values	n	median	IQR	n	median	IQR	*p*-values
Overall score	67	69	54.8–85.7		67	71	57.1–85.7		67	71	50.0–85.7	67	71	42.9–85.7	0,3514
Measure category															
Critical	10	60	53.6–83.3		10	65	60.0–71.4		10	69	50.0–85.7	10	58	50.0–66.7	0.0365*
Major	20	74	55.5–88.7		20	85	71.4–87.5		20	71	57.1–85.7	20	75	57.1–86.6	0,6622
Minor	37	69	56.3–83.3		37	71	50.0–85.7		37	71	42.9–83.3	37	71	37.5–87.5	0,7734
Measure type															
Objective	42	78	71.4–100	< 0.0001*	42	73	64.3–87.5	0.0017*							
Subject	25	50	37.5–66.7		25	56	38.1–78.6								
Domains															
Premises	29	63	50.0–81.3		29	67	57.1–83.3		29	75	50.0–83.3	29	60	37.7–83.3	0.0367*
Dispensing practices	19	75	50.0–91.7		19	75	57.1–100		19	75	62.5–87.5	19	75	37.5–100	0,1140
Store management	15	69	57.1–83.3		15	71	71.4–100		15	57	42.9–71.4	15	71	71.4–85.7	0.0048*
Operations	4	72	38.8–93.8		4	63	50.0–87.5		4	69	52.7–87.5	4	75	25.0–100	0,5775
Level of care															
HC2	66	67	50.0–83.3		65	67	50.0–100		66	67	50.0–100	65	50	33.3–100	0,1959
HC3	66	67	50.0–83.3		66	67	33.3–100		66	67	50.0–100	66	67	33.3–100	0,4131
HC4	67	75	50.0–100		64	100	50.0–100		67	100	50.0–100	67	100	70.9–100	0,9423
n total number of assessments; *IQR* Inter Quartile Range; *p-value < 0.05															
Validity					IRR			Validity							
								Central level Inspector				District based insector			
Adequate IRR and Validity mean score (> 75%)															
	n/N	%	OR (95% CI)		n/N	%	OR (95% CI)		n/N	%	OR (95% CI)	n/N	%	OR (95% CI)	p-values*
Overall > 75%	29/67	43			31/67	46			30/67	45		29/67	43		0,8156
Indicator category															
Critical	4/10	40	1,00		2/10	20	1,00		5/10	50	1,00	2/10	20	1,00	0,1596
Major	10/20	50	1.50 (0.32–6.99)		13/20	65	7.43 (1.23–45.01)**		8/20	40	0.67 (0.15–3.07)	11/20	55	4.89 (0.82–29.06)	0,3422
Minor	15/37	41	1.02 (2.5–4.25)		16/37	43	3.05 (0.57–16.36)		17/37	46	0.85 (0.21–3.44)	16/37	43	3.05 (0.57–16.36)	0,7951
Domains															
Premises	12/29	41	1,00		12/29	41	1,00		15/29	52	1,00	10/29	35	1,00	0,1662
Dispensing practices	10/19	53	1.57 (0.49–5.05)		10/19	53	1.57 (0.49–5.05)		10/19	53	1.04 (0.33–3.30)	10/19	53	2.11 (065–6.89)	1,0000
Store management	5/15	33	0.71 (0.19–2.61)		7/15	47	1.24 (0.35–4.35)		3/15	20	0.23 (0.05–1.01)	7/15	47	1.66 (0.47–5.93)	0,1172
Operations	2/4	50	1.42 (0.17–11.51)		2/4	50	1.42 (0.17–11.51)		2/4	50	0.93 (0.12–7.55)	2/4	50	1.90 (023–15.58)	0,7762
Level of care															
HC2	33/66	50	1,00		25/65	39	1,00		23/66	35	1,00	27/65	42	1,00	0,4103
HC3	30/66	46	0.84 (0.42–1.65)		28/66	42	1.11 (0.55–2.27)		24/66	36	1.11 (0.55–2.27)	28/66	42	0.84 (0.42–1.65)	0,4798
HC4	37/67	55	1.27 (0.64–2.51)		48/64	75	4.32 (2.07–9.06)**		35/67	52	4.32 (2.07–9.06)*	*39/67	58	1.27 (0.64–2.51)	0,4851

n Number of assessments with acceptable IRR or validity score; N total number of assessments: *Two -Sample test of proportions; **p-value < 0.05;

### Validity

Overall, the median validity for all 67 GPP measurements was 69% with 29 (43%) measures having adequate validity scores of ≥75%. Median validity did not differ significantly by indicator category, domain, or level of care. Validity score was highest for major indicators followed by minor and critical indicators, at 74, 69, and 60%, respectively. The domain score only varied slightly, with the highest for the dispensing practices domain at 75%, the lowest for the premises domain (63%), and the level of care score highest for HC 4 and lowest for HC 2—75% versus 67%.

There were no significant differences in percentage of measures having adequate validity (≥75%) by categories (40–50%), domain (33–53%), or level of care (46–55%).

There were no significant differences between district- and central-level inspectors in validity scores overall, or by indicator categories, domain, or level of care scores. Central-level inspectors, compared to district inspectors had significantly higher validity scores for critical indicators (69% versus 58%) and the premises domain (75% versus 60%); whereas, district inspectors scored higher for stores management (71% versus 57%).

### IRR

The median IRR for all GPP measures was 71% with 31 (46%) of the measures having adequate scores of ≥75%. There was no significant difference in median IRR scores by category, domain, or level of care, though the major indicators had the highest category score. The percentage of measures with an adequate IRR differed significantly by indicator category, with major indicators having significantly higher percentage IRR scores (*P* < 0.029) compared to critical indicators, and by level of care, with HC 4 facilities having higher percentages than HC 2 facilities (75% versus 39%, *p* < 0.001). The percent of measures with adequate IRR did not differ significantly by domain. We found that objective measures had a significantly higher IRR (*p* < 0.0001) and validity (*p* = 0.0017) than subjective measures.

**Fig. 2:**
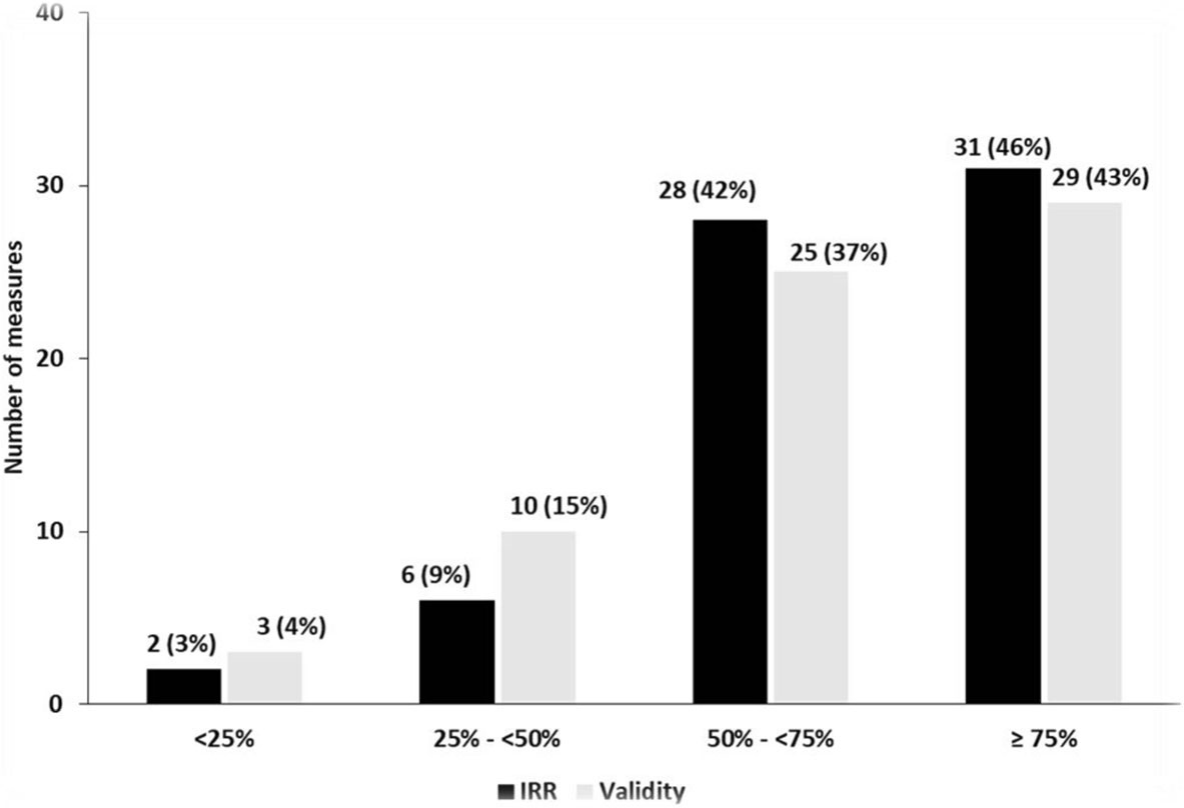
Distribution of GPP measures by Validity and IRR cut offs

Figure 2 shows the distribution of the validity and IRR scores for the 67 GPP measures. Almost half of all measures had adequate (≥75%) IRR scores and slightly fewer had adequate validity scores; 80% or above of the measures had at least moderately adequate (i.e., ≥50%) IRR and validity scores.

### Certification validity score

The gold standard and central-level inspectors arrived at the same inspection outcome for all eight facilities, for a certification validity of 100%. In the eight facilities, the district inspectors did not certify one facility certified by the gold standard inspector (i.e., false negative) and did certify four facilities not certified by the gold standard inspector (i.e., false positive), for a certification validity of 37.5% (i.e., three out of eight) (Table 4). In all four false-positive facilities, only one critical measure per facility differed from the gold standard, which were from the premises domain.

**Table 4:** Validity of certification decision by inspector type

		Gold Standard	
		Certified	Not certified
Central level inspectors	Certified	1	0
	Not Certified	0	7
District based inspectors	Certified	0	4
	Not Certified	1	3

## Discussion

This study measured validity and IRR for the 67 GPP measures that constitute the NDA inspection tool for certifying GPP implementation in public sector health facilities and the validity of certification decisions. The GPP inspection tool uses well-known measures, was thoroughly piloted, and is used by experienced inspectors trained in it. Despite this preparation, we found that median validity and IRR scores for all GPP measures were 69 and 71% respectively, and almost half of the measures achieved adequate (≥75% agreement) validity and IRR scores. Study inspectors were more likely to agree with each other (i.e., IRR) than with the gold standard (i.e., validity). Our findings confirm that validity and IRR must be evaluated when applying an indicator-based assessment method, even when using well-known and globally applied GPP measures and trained inspectors.

Agreement between inspectors and the gold standard was equally weak at all levels of care, for all indicator categories (critical, major, and minor), and all domains. We found that IRR was lowest for critical measures that are essential to facility certification and in lower level of care facilities. This pattern might indicate that as GPP inspection was introduced for the first time in public sector facilities in Uganda, some assessors were more lenient and perhaps more realistic in their judgement of critical measures, especially in lower-level facilities that have limited resources to rectify deficiencies and by particularly district inspectors who had experienced the issues first hand and were cognisant of the resource limitations in their own districts. The adequate validity results confirm this hypothesis—district-based inspectors only agreed with the gold standard inspector on 20% of the critical measures (vs. 50% of the central-based inspectors).

Central-level inspectors had slightly higher adequate overall validity scores for GPP measures and median scores for critical measures, and their premises domain scores were significantly higher than those of district inspectors. Both critical indicators and premises scores greatly influence the certification outcome. The difference in validity of certification decisions between district and central inspectors is a serious concern that will need to be addressed if the certification scheme is to be trusted by the public; in addition, the high rate (50%) of false- positive certifications among district inspectors raises serious public health concerns. The central-level inspectors are generally more engaged in drug regulation and experienced in inspection compared to the district-level inspectors.

For inspections to be trustworthy, it is critical that all inspectors use and interpret the measures in a similar manner, have the same approach, and apply the same judgment. Interventions to simplify tools and further training of inspectors have been associated with improved IRR for good pharmacy practice measures [7]. Applying well-tested, highly uniform, and simple tools increases reliability. In this study, we found the highest IRR and validity scores in the dispensing practices and operations domain. We also found that both IRR and validity of objective measures had a significantly higher rank compared to subjective measures that require personal judgement. It is important to have this in mind when designing measures and indicators. More objective indicators make for a stronger assessment tool, while subjective measures need to be supported by detailed guidelines and training in their practical use.

The operations domain contains the fewest measures (4), and the measures are all binary. Simple binary indicators also had the highest IRR among SPARS measures [7].

The reason for the high score in the dispensing domain is not known. It will be important to improve inspectors’ understanding of GPP measures that had inadequate validity and IRR and consider making changes to simplify and clarify the GPP tool.

Less than a fifth of the GPP measures had IRR and validity scores below 50%. Increasing understanding of and training in these GPP measures will also be critical. Training assessors and strengthening indicator understanding were effective in increasing the IRR of medicines management indicators, resulting in a reduction of indicators having < 50% IRR score from 29 to 4% [7]. Identifying and improving measures that need more clarification and common understanding is an iterative process that need to be continued.

Understanding and interpretation of the GPP measures are slightly better among inspectors than between the inspectors and the gold standard. This finding indicates the need for establishing a common understanding of certain measures among inspectors.

## Limitations

The study has a number of limitations. Since each team had one district- and one central-level inspector, we were unable to assess and compare IRR between two central inspectors and between two district inspectors. Given that the two inspector’s types have different levels of training and experience, we would expect that IRR would be higher among two inspectors of the same type. The study is also limited by the small number of inspections, which made more advanced statistical analysis on correlation between variables underpowered and unfeasible. Some of the statistical tests are underpowered; therefore, even many large differences are not statistically significant.

Another possible limitation is how we handled the missing assessment of critical indicators. In view of the already limited sample size, we did not exclude the measure totally, but we calculated IRR based on seven facilities and validity based on only one inspector compared to the gold standard providing the largest basis for each GPP score calculation. The certification decision was made by giving the missing critical indicators passing scores as the fault is with the inspector and not the facility. This manner of scoring might have slightly improved the certification rate. To increase completeness of the GPP measures and thereby inspection quality, NDA should institute a quality assurance and completeness check following each inspection in both public and private sector inspections.

We chose to measure IRR and validity between two raters using percent agreement because we did not have a sufficient number of facilities per inspector pair to calculate kappa coefficient [9, 10]. Compared to other IRR methods, the percent agreement approach tends to overestimate IRR due to chance agreement. To strengthen our approach, we applied a gold standard inspector to measure validity. We assume the gold standard inspector’s ratings are accurate and give the correct score.

We limited the study to health centres (not hospitals) as they constitute the majority of public sector health facilities and were found to have similar GPP certification rates and IRR scores in the medicines management IRR assessment [7]. Therefore, our results may not be generalizable to hospital settings, which have much higher GPP certification rates.

## Conclusion

NDA must have access to reliable inspection information to ensure quality pharmaceutical services in public and private medicines outlets in Uganda, and NDA has taken an important step to implement GPP inspection in public health facilities. It is critical, however, that licensing and certification decisions are valid and reproducible. This study is the first to report on validity and IRR of 67 GPP measures, finding median validity and IRR to be 69 and 71% respectively, with 43 and 46% percent of indicators achieving adequate score (≥75%). The low validity and IRR of the GPP indicators brings into question some of the inspection outcomes such as certification decisions. NDA will need to apply multipronged interventions to strengthen the validity and reliability of the GPP measures and ensure that the facility certification results are valid.

## Additional files

Additional file 1: ᅟ (PDF 647 kb)

Additional file 2: ᅟ (PDF 237 kb)

## Abbreviations

GPP: Good pharmacy practices
HC: Health centers
IRR: Inter-rater reliability
NDA: National Drug Authority
SPARS: Supervision performance assessment and recognition strategy
