# Supplementary material for: Inter-rater reliability and validity of good pharmacy practices measures in inspection of public sector health facility pharmacies in Uganda
Source: J Pharm Policy Pract. 2019 Jan 22;12:2. doi: 10.1186/s40545-018-0161-y (PMC6341575; doi:10.1186/s40545-018-0161-y)
Supplement: Supplementary file 1 — ᅟ (PDF 647 kb) [file 40545_2018_161_MOESM1_ESM.pdf]

National Drug Authority
P.O BOX 23096 KAMPALA
PLOT 46-48 LUMUMBA AVENUE
Tel. 0414 255665/347391/347392
Fax: 0414 255758
E-mail: ndaug@nda.or.ug; inspectorate@nda.or.ug

INSPECTION REPORT FOR PHARMACY/DRUG OUTLETS IN PUBLIC AND PRIVATE NOT FOR PROFIT (PNFP) HEALTH FACILITIES

Indicator Key:

∗∗	CRITICAL Indicator
∗	MAJOR Indicator
(no stars)	MINOR Indicator

PART A: Pharmacy/drug outlet information

1. NDA Region		
2. District		
3. Name of health facility		
4. Level and ownership (health facility)	Public ○<br>PNFP ○	Hospital ○ HC4 ○ HC3 ○ HC2 ○
a) Physical address of premises		
b) GIS coordinates		
c) Name of in-charge of the facility		
d) Telephone number (for In-Charge)		
e) Email address (for In-Charge)		
5. Is the license application new or renewal (tick)?	Renewal ○ New ○	
a) Write previous license number, if renewal (Indicate N/A for new inspection)		
6. Date of inspection		
7. Name and address of nearest retail pharmacy to premises		
8. Approximate distance to nearest pharmacy (km)		
9. Inspector (name and phone number)		

PART B: Ownership and staffing

<table>
<tr><td>10. Name of owner</td><td colspan="3"></td></tr>
<tr><td colspan="4">11. Number and qualification of staff working in store, pharmacy or dispensary (fill out table below)?</td></tr>
<tr><th>Number of staff</th><th>Qualification</th><th colspan="2">Role (include all roles of staff with this qualification)</th></tr>
<tr><td></td><td></td><td colspan="2"></td></tr>
<tr><td></td><td></td><td colspan="2"></td></tr>
<tr><td></td><td></td><td colspan="2"></td></tr>
<tr><td></td><td></td><td colspan="2"></td></tr>
<tr><td></td><td></td><td>Name</td><td>Qualifications</td></tr>
<tr><td colspan="2">12. Name and qualifications of person dispensing today</td><td></td><td></td></tr>
<tr><td colspan="2" rowspan="2">13. Is the person dispensing today qualified to dispense?</td><td>Yes</td><td></td></tr>
<tr><td>No</td><td></td></tr>
</table>

PART C: Premises

<table>
<tr><th>Indicator</th><th>Location</th><th>Acceptable</th><th>Needs Improvement</th><th>Unacceptable</th></tr>
<tr><td rowspan="2">14. ** Walls are clean without holes and signs of humidity and degradation, and made of a material that allows for easy cleaning (tiles or oil paint).</td><td>Dispensary</td><td></td><td></td><td></td></tr>
<tr><td>Store</td><td></td><td></td><td></td></tr>
<tr><td colspan="5">Comments</td></tr>
<tr><td rowspan="2">15. ** Roof is in good condition to avoid water penetration. It is without holes or signs that water is running through. There is a ceiling made of cardboards, papyrus or cement.</td><td>Dispensary</td><td></td><td></td><td></td></tr>
<tr><td>Store</td><td></td><td></td><td></td></tr>
<tr><td colspan="5">Comments</td></tr>
<tr><td rowspan="2">16. * Floors are made of a material that is easy to clean, free of holes and big cracks and the floor appears clean and newly swept and washed also in the corners.</td><td>Dispensary</td><td></td><td></td><td></td></tr>
<tr><td>Store</td><td></td><td></td><td></td></tr>
<tr><td colspan="5">Comments</td></tr>
<tr><td rowspan="2">17. ** The medicines are protected from direct sunlight (painted glass, curtains, blinds or no windows).</td><td>Dispensary</td><td></td><td></td><td></td></tr>
<tr><td>Store</td><td></td><td></td><td></td></tr>
<tr><td colspan="5">Comments</td></tr>
</table>

**Indicator**	**Location**	**Acceptable**	**Needs Improvement**	**Unacceptable**
18. * There are no signs of pests, harmful insects or rodents seen.	Dispensary			
	Store			
**Comments**				
19. The temperature is monitored daily and the temperature is recorded.	Dispensary			
	Store			
**Comments**				
20. The temperature can be regulated with ventilation, air-condition or by opening windows.	Dispensary			
	Store			
**Comments**				

**Indicator**	**Location**	**Size (m^2)**	**Yes**	**No**
21. Write size (m^2) and score. Space requirements:<br>• dispensary/pharmacy ≥ $4m^2$,<br>• store (drug outlets in HC2/HC3) ≥ $6m^2$ and<br>• store(pharmacies in HC4/hosp) ≥ $20m^2$	Dispensary			
	Store			
**Comments**				

**Indicator**	**Location**	**Acceptable**	**Needs Improvement**	**Unacceptable**
22. * The size of the store is adequate having enough shelves and layout so medicines can be organized systematically.	Dispensary			
	Store			
**Comments**				
23. * The pharmacy/store is lockable and access limited to authorized personnel.	Dispensary			
	Store			
**Comments**				

**Indicator**	**Location**	**Yes**	**No**
24. There are procedures for access when store in-charge is not there?	Dispensary		
	Store		
**Comments**			

Indicator	Location	Acceptable	Needs Improvement	Unacceptable
25. The premises appear clean and tidy. Cleaning of floors is done daily and shelves are dusted weekly. The practices are documented.	Dispensary			
	Store			
**Comments**				
26. Fire safety equipment (fire extinguisher or bucket with sand or water, or big blanket) is available and accessible.	Health facility			
**Comments**				

Indicator	Location	Acceptable	Needs Improvement	Unacceptable	N/A
27. ** There is a functioning system for cold storage/ refrigerator. (Check "N/A" if it is a HC2 with no need for cold system)	Health facility				
**Comments**					
28. The temperature of the refrigerator is monitored and recorded on a daily basis. (Check "N/A" if there is no fridge)	Health facility				
**Comments**					

Indicator	Location	Acceptable	Needs Improvement	Unacceptable
29. **Toilet facilities for staff are acceptable (pit latrine, flush toilet);<br>• hygienic (clean),<br>• functioning (in working condition) and<br>• toilet paper is available to staff.	Health facility or Staff Quarters			
**Comments**				
30. **Hand washing facilities for staff are acceptable;<br>• hygienic,<br>• functioning, and<br>• soap is available	Health facility			
**Comments**				

PART D: Dispensing

Indicator	Location	Acceptable	Needs Improvement	Unacceptable
31. *Appropriate packaging material for tablets and capsules available.	Dispensary			
**Comments**				
32. *Tablet counting tray and spatula/spoon are available. Comment if creative trays are used.	Dispensary			
**Comments**				

Indicator	Location	Yes	No
33. Tablets are not counted with bare hands but using a counting tray, spatula or gloved hands.	Dispensary		
**Comments**			

Indicator	Location	Acceptable	Needs Improvement	Unacceptable
34. Check if tablet counting tray and spatula and/or gloves are clean.	Dispensary			
**Comments**				
35. There is provision for cleaning utensils with clean water.	Health facility			
**Comments**				
36. All tins/bottles that have been opened but are not in use (no current dispensing from it) are covered with a lid.	Dispensary			
**Comments**				
37. There is a control of the prescription carried out before dispensing (right medicine, strength and dose appropriate for indication and patient age) (prescription control).	Dispensary			
**Comments**				

Indicator	Location	Acceptable	Needs Improvement	Unacceptable
38. There is a control to countercheck the medicine to be dispensed (medicine, strength and quantity correct according to prescription) (dispensing control)?	Dispensary			
**Comments**				
39. Privacy is achieved during dispensing (other waiting patients should be at least 2 meters away from dispensing window).	Dispensary			
**Comments**				
40. Chairs/benches available for customers/patients so they can sit while waiting for their medicines?	Dispensary			
**Comments**				
41. Hand washing facilities and soap available to customer/patient.	Dispensary			
**Comments**				
42. Drinking water (to take tablets) is available to customer/patient (the water should be bottled, purified or boiled).	Dispensary			
**Comments**				

Indicator	Location	Yes	No
43. *A prescription recording system is available.	Health facility		
**Comments**			

Indicator	Location	Acceptable	Needs Improvement	Unacceptable
44. *Prescription recording system includes date, patient number or name, diagnosis, medicine name, prescriber name, amount prescribed and dispensed.	Health facility			
**Comments**				

**Indicator**	**Location**	**Acceptable**	**Needs Improvement**	**Unacceptable**	**N/A**
45. A book for prescription for class A drugs (narcotics) are available in HC IV and hospitals. (Select N/A for HC III and HC II.)	Health facility				
**Comments**					

**Indicator**	**Location**	**Yes**	**No**
46. Checks that prescriptions/prescription books (can be the OPD book) are kept for a minimum of 5 years?	Health facility		
**Comments**			
47. Observe that ALL patients receiving dispensed prescription medicines are recorded	Health facility		
**Comments**			

		**Acceptable**	**Needs Improvement**	**Unacceptable**
48. **Medicines are labeled correctly. Check if the labeling on the dispensing envelope is proper for 3 patients coming from pharmacy. Fill in the table below with Yes or No.<br>• Select "Acceptable" if all areas in columns 1)-6) are scored yes.<br>• Select "Needs Improvement" if at least all areas in columns 1) and 4) are scored yes.<br>• Select "Unacceptable" if there is a single "No" score in any area under columns 1) and 4)	Health facility			

**1) Medicine name**	**2) Strength**	**3) Quantity**	**4) Dosage (dose)**	**5) Patient name**	**6) Facility name**

Comments

49. Customer/patient knows how to take medicine. Check by asking 3 patients (the same as labeling is checked for) coming from pharmacy and fill the table below indicating if patient knows 'Yes' or does not know 'No'.<br>• Select "Yes" if at least all areas in columns 1) 2) 5) scored yes.<br>• Select "No" if any areas in Columns 1) 2) 5) has a no	Health facility	Yes	No

**1) Dose (how much to take)**	**2) Frequency (how often to take)**	**3) Duration (how long to take)**	**4) Indication (why to take)**	**5) Instructions on how to take medicine (with food etc)**

Comments

PART E: Store management

**Indicator**	**Location**	**Acceptable**	**Needs Improvement**	**Unacceptable**
50. * Medicine packs are stored only on shelves and/or in cupboards and they are not stored in disorganized stacks or boxes directly on the floor	Dispensary			
	Store			
**Comments**				
51. * Stock cards are available (check for 10 items).	Store			
**Comments**				

**Indicator**	**Location**	**Acceptable**	**Needs Improvement**	**Unacceptable**	**N/A**
52. * Stock cards are kept next to the medicines on shelves (or organized in a folder in the store). Check to see.	Store				
**Comments**					

Indicator	Location	Acceptable	Needs Improvement	Unacceptable	N/A
53. Stock card headers are filled correct with medicine name, strength, dosage form, special storage (check 10 items)? Score 'Acceptable' if all 10 stock cards are correct.	Store				
**Comments**					
54. Physical count is done monthly and clearly indicated in stock cards. Check for 5 items.	Store				
**Comments**					
55. Stock cards correctly updated so physical count and stock card balance is the same (check for 5 items).	Store				
**Comments**					

Indicator	Location	Acceptable	Needs Improvement	Unacceptable
56. Medicine is stored in a systematic manner (alphabetic or therapeutic)?	Dispensary			
	Store			
**Comments**				

Indicator	Location	Acceptable	Needs Improvement	Unacceptable
57. Shelves are labeled with medicine name or class?	Dispensary			
	Store			
**Comments**				

58. Is pharmacy/drug outlet computerized?	Health facility	**Yes**	
		**No**	
**Comments**			

Indicator	Location	Acceptable	Needs Improvement	Unacceptable
59. *There is a record for expired/damaged medicines and health supplies including name, strength, formulation, batch number, expiry date and quantity (check).	Store			
**Comments**				

Indicator	Location	Acceptable	Needs Improvement	Unacceptable
60. * There is a designated area to store expired/damaged medicines and health supplies away from useable medicine, and the area is clearly marked/ signed?	Store			
**Comments**				
61. * First Expiry First Out (FEFO) is adhered to (check 10 randomly selected medicine/health supplies)?	Store			
**Comments**				

PART F: Operating requirements

Indicator	Location	Yes	No	N/A
62. Certificate for suitability in Good Pharmacy Practice displayed	Health facility			
**Comments**				
63. The latest version of reference material available<br>• Essential Medicines and Health Supplies List for Uganda<br>• Uganda Clinical Guidelines (current edition)<br>(If at least one of the materials is available, check Yes)<br>Comment if the materials are not available at pharmacy/dispensary	Health facility	Yes		
		No		
**Comments**				

Indicator	Location	Acceptable	Needs Improvement	Unacceptable
64. Does the drug outlet have written procedures for the following<br>• Dispensing<br>• Receipt of goods<br>• Stock management	Health facility			
**Comments**				
65. *Records (includes delivery notes) kept for received items. Check for 10 items.	Health facility	Yes		
		No		
**Comments**				

66. Do the records include:	**Yes**	**No**
a) Date of receipt		
b) Invoice number		
c) Origin (producer/supplier)		
d) Quantity received		
e) Batch number		
f) Expiry date		
**Comments**		

PART G: Summary of findings

Count the number of “unacceptable “/”No” scores

	Number
**CRITICAL Indicators with a score of "Unacceptable"/"No"	
*MAJOR Indicators with a score of "Unacceptable"/"No"	
MINOR Indicators with a score of "Unacceptable"/"No"	

Drug outlet falls into the following category (check ***one***) (Adhere to the certification criteria)

Certification without comments	
Certification with minor comments	
Certification with major comments	
Not certified until improvements are made	

GPP certification number/license number (only if GPP certification is given):

____

Improvement is needed for the following indicators (see table below)

To get GPP certification Yes ○ No ○

To keep GPP certification Yes ○ No ○

Indicator Number	Inspector's Observation

Indicator Number	Inspector's Observation

PART H: Signatures

The inspector signing below **declares that all the above corresponds with the actual findings** in the pharmacy on the day of the inspection

_____________________________ _______________________ _______________________
Name of Inspector Designation Signature

This is **confirmed** by the signature of the Head, Drug Inspectorate Services

____________________________________ _____________ __________________________
Name of Head, Drug Inspectorate Services Date Signature
