# Supplementary material for: Inter-rater reliability and validity of good pharmacy practices measures in inspection of public sector health facility pharmacies in Uganda
Source: J Pharm Policy Pract. 2019 Jan 22;12:2. doi: 10.1186/s40545-018-0161-y (PMC6341575; doi:10.1186/s40545-018-0161-y)
Supplement: Supplementary file 2 — ᅟ (PDF 237 kb) [file 40545_2018_161_MOESM2_ESM.pdf]

Additional file 2: Overall mean IRR and validity % for GPP measures in Uganda.

Type*	Domain**	Mean score of GPP measures	Validity %			IRR %
			Inspector			
			District	Central	Overall	Overall
		**Critical Measures**				
s	P	Wall are clean-dispensary	50%	63%	56%	63%
s	P	Wall are clean-store	67%	50%	58%	60%
s	P	Roof is in good condition-dispensary	50%	75%	63%	63%
s	P	Roof is in good condition-store	67%	100%	83%	67%
o	P	Medicines protected from sun-dispensary	50%	57%	54%	71%
o	P	Medicines protected from sun-store	100%	83%	92%	100%
o	P	There is a functioning cold storage system	80%	100%	90%	83%
s	P	Toilets for staff are acceptable	14%	43%	29%	38%
s	P	Hand washing facilities are acceptable	0%	38%	19%	29%
o	D	Medicines are labelled correctly	67%	86%	76%	67%
		Mean score for critical measures	***54%***	***69%***	***62%***	***64%***
		Number of critical measures with acceptable score (≥75%) # (%)	***2 (20%)***	***5 (50%)***	***4 (40%)***	***2 (20%)***
		**Major Measures**				
s	P	Floors made materials easy to clean-dispensary	88%	75%	81%	88%
s	P	Floors made materials easy to clean-store	33%	43%	38%	83%
s	P	No signs of pests-dispensary	38%	50%	44%	38%
s	P	No signs of pests-store	33%	60%	47%	50%
s	P	Store size is adequate with enough shelves and layout-dispensary	43%	43%	43%	75%
s	P	Store size is adequate with enough shelves and layout-store	86%	71%	79%	86%
s	P	Pharmacy/store lockable and limited access-dispensary	71%	86%	79%	75%
s	P	Pharmacy/store lockable and limited access-store	86%	100%	93%	86%
o	D	Appropriate packaging materials available	100%	100%	100%	100%
o	D	Tablet counting tray and spatula are available	75%	63%	69%	88%
o	D	Prescription recording system is available	100%	100%	100%	100%
o	D	Prescription recording system data is available	75%	63%	69%	63%
o	S	Medicine packs are stored only on shelves and cupboards - dispensary	20%	33%	27%	67%
o	S	Medicine packs are stored only on shelves and cupboards - store	80%	71%	76%	100%
o	S	Stock cards are available - store	100%	83%	92%	71%
o	S	Stock cards are kept next to the medicines on shelves - store	71%	71%	71%	86%
o	S	There is record of expired/damaged medicines and health supplies - store	86%	86%	86%	71%
o	S	There is a designated area to store expired/damaged medicines - store	71%	57%	64%	86%
o	S	FEFO is adhered to - store	71%	57%	64%	71%
o	O	Records kept for received items - HF	100%	100%	100%	100%
		Mean score for major measures	***71%***	***71%***	***71%***	***79%***
		Number of major measures with acceptable score (≥75%) # (%)	***11 (55%)***	***8 (40%)***	***10 (50%)***	***13 (65%)***

Type*	Domain**	Mean score of GPP measures	Validity %			IRR %
			Inspector			
			District	Central	Overall	Overall
		**Minor Measures**				
o	P	Temperature is monitored-dispensary	29%	29%	29%	75%
o	P	Temperature is monitored-store	67%	50%	58%	60%
o	P	Temperature can be regulated-dispensary	86%	57%	71%	71%
o	P	Temperature can be regulated-store	80%	83%	82%	75%
o	P	Space requirements OK-dispensary	83%	80%	82%	67%
o	P	Space requirements OK-store	100%	80%	90%	83%
s	P	Procedure to access office when store in-charge is absent-dispensary	38%	63%	50%	25%
s	P	Procedure to access office when store in-charge is absent-store	33%	83%	58%	17%
s	P	Premises are clean and tidy-dispensary	38%	75%	56%	63%
s	P	Premises are clean and tidy-dispensary-store	60%	100%	80%	50%
o	P	Fire safety equipment is available	57%	86%	71%	57%
o	P	Temperature of the refrigerator is monitored and recorded daily	75%	88%	81%	88%
o	D	Tablet are not counted with bare hands but with counting tray	63%	75%	69%	63%
s	D	Counting trays and spatula or gloves are clean	13%	38%	25%	50%
o	D	Provision for cleaning utensils with clean water	14%	25%	20%	29%
s	D	Tins/bottles that have been opened are covered with lid	71%	88%	79%	57%
s	D	There is control of the prescription before dispensing	25%	38%	31%	13%
s	D	There is control to countercheck the medicines dispensed	14%	43%	29%	38%
o	D	Privacy is achieved during dispensing	50%	71%	61%	86%
o	D	Chairs/benches are available for patients	100%	100%	100%	88%
o	D	Hand washing facilities and soap available to patients	38%	63%	50%	63%
o	D	Drinking water is available to patients	88%	88%	88%	75%
o	D	A book for prescription for class A drugs available	100%	100%	100%	100%
o	D	Prescription books are kept for minimum 5 yrs.	88%	88%	88%	100%
o	D	All patients receiving dispensed prescription medicine are recorded	83%	67%	75%	100%
o	D	Customers know how to take medicines	100%	83%	92%	100%
o	S	Stock cards headers are filled correct with medicine data - store	71%	71%	71%	100%
o	S	Physical count done monthly and clearly indicated on card - store	71%	43%	57%	71%
o	S	Stock cards correctly updated so physical count and stock card balance is same - store	71%	43%	57%	71%
o	S	Medicine is stored in a systematic manner - dispensary	100%	33%	67%	50%
o	S	Medicine is stored in a systematic manner - Store	67%	43%	55%	100%
s	S	Shelves are labelled with medicine names or class - dispensary	100%	67%	83%	50%
o	S	Shelves are labelled with medicine names or class - store	80%	57%	69%	80%
o	S	Is the pharmacy or drug outlet computerised	86%	86%	86%	100%
o	O	Latest reference material available - HF	100%	75%	88%	75%
s	O	Does the drug outlet have written procedures - FH	0%	43%	21%	50%
s	O	Do the records include key qualities	50%	63%	56%	50%
		Mean score for minor measures	***65%***	***67%***	***66%***	***67%***
		Number of minor measures with acceptable score (≥75%) # (%)	***16 (43%)***	***17 (46%)***	***15 (41%)***	***16 (43%)***
**Mean score for all measures**			***65%***	***68%***	***67%***	***70%***
**Total number of measure with acceptable score (≥75%)**			***29 (43%)***	***30 (45%)***	***29 (43%)***	***31 (46%)***

*** o= Objective; s= subjective; ** P= Premises; D=Dispensing practice; S=Store management; O=Operations**

Mean score of GPP measures	Validity %			IRR %
	Inspector			
	District	Central	Overall	Overall
**Domain**				
**Premises *(n = 29)***				
Mean score for premises domain measures	59%	69%	64%	65%
Number of critical measures with acceptable score (≥75%) # (%)	*10 (35%)*	*15 (52%)*	*12 (41%)*	*12 (41%)*
**Dispensing practice (n = 19)**				
Mean score for Dispensing practice domain measures	67%	72%	69%	72%
Number of critical measures with acceptable score (≥75%) # (%)	***10 (53%)***	***10 (53%)***	***10 (53%)***	***10 (53%)***
**Store management (n = 15)**				
Mean score for store management domain measures	76%	60%	68%	78%
Number of critical measures with acceptable score (≥75%) # (%)	***7 (47%)***	***3 (20%)***	***5 (33%)***	***7 (47%)***
**Operations (n = 4)**				
Mean score for operations domain measures	63%	70%	66%	69%
Number of critical measures with acceptable score (≥75%) # (%)	***2 (50%)***	***2 (50%)***	***2 (50%)***	***2 (50%)***
**Level of Care**				
**HC 2 (n = 67)**				
Mean score for HC 2 measures***	59%	65%	62%	67%
Number of critical measures with acceptable score (≥75%) # (%)*	27 (42%)	23 (35%)	33 (50%)	25 (39%)
**HC 3 (n = 67)**				
Mean score for HC 3 measures***	63%	68%	66%	63%
Number of critical measures with acceptable score (≥75%) # (%)*	28 (42%)	24 (36%)	30 (46%)	28 (42%)
**HC 4 (n = 67)**				
Mean score for HC 4 measures***	71%	69%	71%	81%
Number of critical measures with acceptable score (≥75%) # (%)*	39 (58%)	35 (52%)	37 (55%)	48 (75%)
***Missing data is considered in the % calculations				
